# Historical Comparison of Perfluorooctanesulfonate, Perfluorooctanoate, and Other Fluorochemicals in Human Blood

**DOI:** 10.1289/ehp.7544

**Published:** 2005-02-02

**Authors:** Geary W. Olsen, Han-Yao Huang, Kathy J. Helzlsouer, Kristen J. Hansen, John L. Butenhoff, Jeffrey H. Mandel

**Affiliations:** ^1^Medical Department, 3M Company, St. Paul, Minnesota, USA;; ^2^Department of Epidemiology, Johns Hopkins Bloomberg School of Public Health, Baltimore, Maryland, USA;; ^3^George W. Comstock Center for Public Health Research and Prevention, Johns Hopkins University, Baltimore, Maryland, USA;; ^4^Prevention and Research Center, Women’s Center for Health and Medicine, Mercy Medical Center, Baltimore, Maryland, USA;; ^5^Environmental Laboratory, 3M Company, St. Paul, Minnesota, USA

**Keywords:** fluorochemicals, perfluorohexanesulfonate, perfluorooctanesulfonate, perfluorooctanoate, PFHS, PFOA, PFOS

## Abstract

The purpose of this investigation was to determine whether there has been a change in the human blood concentration of perfluorooctanesulfonate (PFOS), perfluorooctanoate (PFOA), and five other fluorochemicals since 1974. Blood samples were collected in 1974 (serum) and 1989 (plasma) from volunteer participants of a large community health study. The study included a total of 356 samples (178 from each time period). These samples were analyzed by high-pressure liquid chromatography/tandem mass spectrometry methods. The median 1974 and 1989 fluorochemical concentrations, respectively, were as follows: PFOS, 29.5 ng/mL vs. 34.7 ng/mL; PFOA, 2.3 ng/mL vs. 5.6 ng/mL; perfluorohexanesulfonate (PFHS), 1.6 ng/mL vs. 2.4 ng/mL; and *N*-ethyl perfluorooctanesulfonamidoacetate (PFOSAA), less than the lower limit of quantitation (LLOQ; 1.6 ng/mL, vs. 3.4 ng/mL). For *N*-methyl perfluorooctanesulfonamidoacetate (M570), perfluorooctanesulfonamide, and perfluorooctanesulfonamidoacetate, median serum concentrations in both years were less than the LLOQ values (1.0, 1.0, and 2.5 ng/mL, respectively). Statistical analysis of 58 paired samples indicated that serum concentrations of PFOS, PFOSAA, PFOA, PFHS, and M570 were significantly (*p* < 0.001) higher in 1989 than in 1974. The data from 1989 were then compared with geometric mean fluorochemical concentrations of serum samples collected in 2001 from 108 American Red Cross adult blood donors from the same region. Except for M570, there were no statistically significant (*p* < 0.05) geometric mean fluorochemical concentration differences between the 1989 and 2001 samples. In conclusion, based on this study population, PFOS and other serum fluorochemical concentrations have increased between 1974 and 1989. Comparison with other regional data collected in 2001 did not suggest a continued increase in concentrations since 1989.

Perfluorooctanesulfonyl fluoride (POSF; C_8_F_17_SO_2_F) was used to synthesize high-molecular-weight fluorochemical products manufactured by the 3M Company (3M). Unique compounds were created by derivatization of POSF through the sulfonyl fluoride moiety using conventional hydrocarbon reactions (3M Company 2003). Although these fluorochemical products tended to be stable in the environment, secondary reactions producing these polymers may have had unreacted or partially reacted starting materials or intermediates that were carried forward to the final product. These fluorochemical residuals were typically present at a concentration of ≤ 1% in the final commercialized products. These residuals could include *N*-methyl perfluorooctanesulfonamidoacetate [M570; C_8_F_17_SO_2_N(CH_3_)CH_2_COO^−^], *N*-ethyl perfluorooctanesulfonamidoacetate [PFOSAA;C_8_F_1 7_SO_2_N(CH_2_CH_3_)CH_2_COO^−^], perfluorooctanesulfonamidoacetate (M556; C_8_F_17_SO_2_NHCH_2_COO^−^), perfluorooctanesulfonamide (PFOSA; C_8_F_17_SO_2_NH_2_), perfluorooctanoate (PFOA; C_7_F_15_COO^−^), and perfluorohexanesulfonate (PFHS; C_6_F_13_SO_3_^−^).

M570 is an oxidation product of *N*-methyl perfluorooctanesulfonamidoethanol [N-MeFOSE; C_8_F_17_SO_2_N(CH_3_)CH_2_CH_2_OH], a residual found in products used primarily in surface treatment applications (e.g., carpets, upholstery, textiles). Commercialization of this product line began in the 1950s. PFOSAA is an oxidation product of *N*-ethyl perfluorooctanesulfonamidoethanol [N-EtFOSE; C_8_F_17_SO_2_N(CH_2_CH_3_)CH_2_CH_2_OH], a residual found in products of the phosphate ester of N-EtFOSE that were primarily used in paper and packaging protectant applications. PFOSAA was also sold as a surfactant. Commercialization of this product line began in the late 1960s with the introduction of human food contact paper applications in 1974. Both PFOSAA and M570 are thought to metabolize to M556 and PFOSA. The terminal fluorochemical moiety of the degradation pathway has been shown to be perfluorooctanesulfonate (PFOS; C_8_F_17_SO_3_^−^). Unlike PFOSAA and M570, PFOS is not specific to any consumer application, but could stem from product or environmental exposures.

Although PFOA and PFHS have been analyzed as fluorochemical residuals in POSF-based materials (Wendling 2003), the primary role of these compounds was with specific applications. PFOA was produced by 3M and other manufacturers to be an emulsifier in a variety of industrial fluoropolymer applications. PFOA may also be produced by environmental degradation of some PFOS precursors (Lange 2001) or by oxidation or metabolism of the widely used telomer-based fluorochemicals manufactured by other companies (Hagen et al. 1981). 3M produced PFHS as a building block for compounds incorporated in fire-fighting foams and specific postmarket carpet treatment applications.

In May 2000, 3M announced that it would voluntarily cease manufacturing POSF-based materials after PFOS was found to be widespread in human populations and wildlife [3M Company 2003; Giesy and Kannan 2001; Hansen et al. 2001; Kannan et al. 2001a, 2001b, 2002a, 2002b, 2002c, 2002d; Organisation for Economic Co-operation and Development (OECD) 2002].

The serum concentration of PFOS has been reported to be, on average, 30–40 ng/mL in nonoccupational human serum and liver samples obtained in the United States (Hansen et al. 2001; Olsen et al. 2003a, 2003b, 2004c, 2004d). Geographic differences may exist by country (Kannan et al. 2004). In the largest biomonitoring study of adults in the United States (598 blood donors 20–69 years of age), Olsen et al. (2003c) reported a geometric mean (GM) serum PFOS concentration of 35 ng/mL [95% confidence interval (CI), 33–37 ng/mL]. The upper bound of the 95th percentile estimate was 100 ng/mL. Adjusted for age and sex, there were minimal differences between the six locations from which the samples were obtained. Similar findings for PFOS have been reported in children and the elderly (Olsen et al. 2004a, 2004b). After consideration of serum and liver PFOS concentrations associated with no observed adverse effect levels (NOAEL) and benchmark dose calculations from several toxicologic studies, a human serum concentration of 100 ng/mL PFOS (the upper bound estimate of the 95th percentile observed in the children, adult, and elderly populations studied in the United States described above) was related to margins of exposure ranging from 310 (decreased rat pup weight gain) to 1,550 (liver tumors in adult rats) (3M Company 2003).

Although not fully understood, the pathways leading to the presence of PFOS in serum and tissue in the general population include release to the environment of POSF-based materials in the waste streams generated from the manufacturing processes and supply chain operations, and/or exposures to consumer products (3M Company 2003; Hansen et al. 2002; Martin et al. 2002; OECD 2002). The other six fluorochemicals mentioned above have also been quantified in the general population at concentrations that are approximately an order of magnitude lower than that of PFOS (Olsen et al. 2003c, 2004a, 2004b). In occupationally exposed populations, serum PFOS concentrations have averaged between 500 and 2,000 ng/mL, depending on the type of job (Olsen et al. 2003a, 2003b).

An extensive PFOS-related toxicology database is reviewed elsewhere (3M Company 2003; OECD 2002). Studies in rats show that PFOS is well absorbed orally and distributes primarily in the liver and, to a lesser extent, in the serum/plasma of rats (Johnson et al. 1979). Substantial enterohepatic circulation of PFOS occurs with both urinary and fecal excretion (Johnson et al. 1984). Dermal absorption of PFOS appears to be possible but limited (3M Company 2003). Because of the exceptionally low vapor pressure of PFOS, inhalation exposure to vapor would be unlikely. If inhalation exposure does occur, it would be most likely associated with aerosols or particulates containing PFOS. In the production of PFOS and related chemistries, exposure to the volatile PFOS precursor POSF was possible. In addition to POSF, certain other precursor molecules, such as N-EtFOSE and N-MeFOSE, are known to sublime. These precursors may represent a more likely inhalation exposure pathway than exposure to PFOS itself (3M Company 2003).

Mechanisms of toxicity in the animal studies are not fully understood but may include effects on fatty acid transport and metabolism, membrane function and mitochondrial bioenergetics (Berthiaume and Wallace 2002; Haughom and Spydevold 1992; Hu et al. 2002; Luebker et al. 2002; Starkov and Wallace 2001). Lowered serum total cholesterol appeared to be a consistent early finding, with cumulative toxicity resulting in metabolic wasting and ultimately death in laboratory animals exposed to high doses (Seacat et al. 2002a, 2002b, 2003). Two-year feeding studies of PFOS in rats produced a modest, largely benign, liver tumor response in the high-dose group (20 ppm PFOS in feed), which likely occurred through nongenotoxic mechanisms (Seacat et al. 2002a). PFOS reduced postnatal survival and body weight gains in dams and pups in reproduction and development studies of mice and rats (Lau et al. 2003; Thibodeaux et al. 2003). *In utero* exposure is likely in humans because a high correlation (*r*^2^ = 0.9) of PFOS concentrations (range, 5–18 ng/mL) was shown in 15 maternal and cord blood samples from Japan (Inoue et al. 2004).

As reviewed by Kennedy et al. (2004), ingestion, inhalation, and dermal pathways are potential pathways of absorption for PFOA and there are marked pharmacokinetic differences by species and by sex within some species. PFOA is a peroxisome proliferator and exerts morphologic and biochemical effects characteristic of peroxisome proliferator-α receptor (PPAR) agonists. These effects included increased β-oxidation, increases in several cytochrome P-450–mediated reactions, and inhibition of the secretion of very-low-density lipoproteins and cholesterol from the liver (Kennedy et al. 2004). The triad of tumors observed (liver, Leydig cell, and pancreatic acinar cell tumors) in a lifetime bioassay study of rats fed PFOA is typical of many PPAR agonists and is believed to involve nongenotoxic mechanisms (Biegel et al. 2001; Kennedy et al. 2004; Klaunig et al. 2003). After consideration of serum and liver PFOA concentrations associated with NOAELs and benchmark dose calculations from several toxicologic studies, a human serum concentration of 14 ng/mL PFOA (the upper bound estimate of the 95th percentile observed in the children, adult, and elderly populations studied in the United States described above) was related to margins of exposure between 1,600 (liver weight increase in rats) to 8,900 (Leydig cell adenoma) (Butenhoff et al. 2004).

Although current serum PFOS and PFOA concentrations have been characterized (Olsen et al. 2003c, 2004a, 2004b), few samples have been analyzed to determine temporal trends in serum concentrations in the general population (3M Company 2003). In one analysis, PFOS was not detected in 10 pooled samples (10 donors per sample) from U.S. military recruits of the Korean War era, a period just before the commercial introduction of POSF-based materials (3M Company 2003). The purpose of the present investigation was to assess whether blood concentrations of PFOS, PFOA, and five other fluorochemicals may have increased during a 27-year time period in a selected sample of a general population. Repository blood samples were obtained from participants of a community study conducted in 1974 and 1989 in Washington County, Maryland (Comstock et al. 1988, 1997), whose county seat is Hagerstown. These findings were then compared with 108 American Red Cross adult blood donor samples collected from the Hagerstown area in 2001 whose GM serum level was 35 ng/mL (95% CI, 32–39) with a range of 8–226 ng/mL (Olsen et al. 2003c).

## Materials and Methods

### Study population.

Blood samples were obtained from two large community-based cohorts established in Washington County, Maryland, in 1974 and 1989 for studying clues to the development of heart disease and cancer. The volunteer participants were asked to donate a blood sample and respond to a brief questionnaire that inquired about demographic characteristics and health-related variables. Among the donors, there were 5,497 and 2,172 participants in 1974 and 1989, respectively, who were not in the subsequent analytical cohort (residents within a 30-mile radius of the study center), and the samples were eligible to be used for the present study. Most of these participants were from geographic communities contiguous to Washington County in Maryland, Pennsylvania, West Virginia, or Virginia. The present study was approved by the Committee on Human Research of the Johns Hopkins Bloomberg School of Public Health.

The serum samples (collected in 1974) and plasma samples (collected in 1989) of these out-of-county residents were stored at −70°C until thawed for the present study. A total of 356 blood samples (178/year) were randomly selected across sex and 10-year age strata. Fifty-eight individuals participated in both years providing paired samples. These 58 individuals were selected to examine within-person change levels with a statistical power of 85% to detect a 10% change between 1974 and 1989. The nonpaired samples (120/year) were selected with 80% statistical power to detect a 20% change, stratified by sex and age groups of < 40, 40–60, and > 60 to ensure equal numbers in each stratum.

### Laboratory assay.

A selective and sensitive method for analysis of PFOS, PFOA, PFHS, PFOSAA, M570, M556, and PFOSA in human serum and plasma, using liquid chromatography/tandem mass spectrometry, was developed and validated (Tandem Labs 1999, 2001a, 2001b). The results of the human serum and plasma validation experiments complied with the precision and accuracy limits (± 15%) (Benson and Ourisson 2001) defined by the U.S. Food and Drug Administration (FDA) guidance document (FDA 2001).

Additionally, serum quality control samples were analyzed against plasma calibration, because plasma has lower endogenous levels of some analytes than does serum. This method of quantitating serum samples against plasma calibration also met the FDA ± 15% method precision requirement for all target analytes and also met the FDA ± 15% method accuracy requirement for PFOS, M556, and M570. Method accuracy for serum analysis against plasma calibration was expanded to ± 19% for PFOA, ± 21% for PFOSAA, and ± 26% for PFHS. Method accuracy for serum analysis against plasma calibration was less acceptable for PFOSA at 43%.

Together, these validations showed that the method is valid for the quantitation of human sera samples using calibration curves in serum, as well as using calibration curves in plasma (Tandem Labs 1999, 2001a, 2001b). Although the use of plasma for the quantitation of sera samples does not strictly meet the FDA requirements for accuracy for the target analytes except PFOS, M556, and M570, the validations showed that the only difference that it introduced was a relatively small systematic bias while maintaining excellent precision. This bias may be corrected for, if desired. This correction was not deemed necessary for the purposes of biomonitoring studies performed by 3M. A validation study of the methods used in the present study also demonstrated stability of the analytes regarding the number of freeze/thaw cycles (Tandem Labs 1999, 2001a, 2001b).

The specific analytical methods used for the analysis of these fluorochemicals in human serum samples have been described previously (Olsen et al. 2003c, 2004a, 2004b) and for the purpose of brevity are not detailed here. A report describing the specific analyses done in the present study is available (Tandem Labs 2001c). Briefly, the method used to analyze serum and plasma samples consisted of a liquid:liquid extraction procedure followed by evaporation and reconstitution of the extract residue with 20 mM ammonium acetate in water:20 mM ammonium acetate in methanol (30:70, vol/vol). The samples were analyzed by liquid chromatography/tandem mass spectrometry using an API 3000 (Applied Biosystems, Foster City, CA). The instrument was operated in the multiple reaction monitoring mode under optimized conditions for the PFOS, PFOA, PFHS, PFOSAA, PFOSA, M556, and M570 detection of the negative ions formed by TurboIonSpray ionization (Applied Biosystems, Foster City, CA). Quantitation of the target analytes in serum samples was performed by comparing the chromatographic peak areas for each compound with those generated in a series of extracted calibration standards prepared from control plasma determined to contain < 4 ng/mL of any of the target analytes. Evaluation of quality control samples injected during the analytical runs of the 356 samples indicated that the reported quantitative results may have varied ± 10% for precision and accuracy (Tandem Labs 2001c). The repeatability of fluorochemical analyses was assessed by nine pairs of identical samples that were randomly inserted into vial arrays for analyses. The intraset coefficients of variation for the fluorochemical measurements on 1974 and 1989 samples ranged from 3–5% and 3–8%, respectively.

The same analytical methods and laboratory were used in both the present investigation and the analysis of the Hagerstown samples collected in 2001 (Olsen et al. 2003c), although the latter were not analyzed concurrently with the present study samples.

### Data analysis.

PFOS was the only fluorochemical for which all samples had concentrations above the lower limit of quantitation (LLOQ). For statistical analysis, values of other fluorochemicals < LLOQ were assigned the midpoint between zero and the LLOQ.

Using the Wilcoxon rank sum test, we assessed whether concentrations of fluorochemicals differed by sex or age (< 40, 40–60, and > 60 years). Among the 58 paired samples, we assessed the changes in fluorochemicals over time using the Wilcoxon signed-rank test. Median regression models were used to assess whether concentrations were different by the two time points, with and without adjustment for sex and age.

Because PFOSAA and M570 are metabolized to M556 and PFOSA and then to PFOS, we explored whether concentrations of the precursor compounds, PFOSAA or M570, correlated with or predicted concentrations of PFOS, using median regression models.

In order to minimize parametric assumptions in the estimation of extreme percentiles of the population, we used the statistical bootstrap method of Efron and Tibshirani (1993) to generate confidence limits around the empirical percentiles for serum concentrations in the two time periods. In this method, a large number of replicated estimates of the percentile are generated from full-size samples of the original observations drawn with replacement. The distribution of the deviations of replicates from the original-sample estimate mimics the underlying sampling distribution for the estimate. Bias-corrected, accelerated percentiles were used to minimize residual bias. The bias correction factor is derived by comparing empirical percentiles with bootstrap percentiles, and acceleration is accomplished by partial jackknifing. Olsen et al. (2003c, 2004a, 2004b) employed identical estimation methodology in their analyses of children, adult, and elderly samples.

## Results

The demographic characteristics of the study participants are presented in Table 1. About half were men and half never smoked cigarettes. The percentages of blood samples in 1974 that were quantifiable along with the medians and interquartile ranges (IQRs), and GMs and 95% CIs, of the fluorochemicals measured are presented in Tables 2–3. Data for PFOSA and M556 are not presented because all samples collected in 1974 were reported at < LLOQ, 1.0 and 2.5 ng/mL, respectively, for these two analytes. Median concentrations of PFOS and PFHS were statistically significantly (*p* < 0.05) higher in men than in women in 1974, whereas no sex-related differences in PFOSAA, PFOA, or M570 concentrations were observed. Median PFOS and PFHS concentrations were significantly lower in individuals younger than 40 years of age (Tables 2 and 3).

The percentages of blood samples in 1989 that were quantifiable along with the medians and IQRs, and GMs and 95% CIs of the fluorochemicals measured are presented in Tables 4 and 5. Data for PFOSA and M556 are also not presented in Tables 4 and 5 because all PFOSA values and 96% of the M556 values were reported at < LLOQ, 1.0 and 2.5 ng/mL, respectively. Median concentrations of PFOS and PFHS were statistically significantly higher in men than in women in 1989, whereas no sex-related differences in PFOSAA, PFOA, or M570 concentrations were observed. Median serum concentrations for PFOA and PFHS were also significantly lower in persons younger than 40 years of age in 1989. Statistically significant differences by age were not observed for PFOS, PFOSAA, or M570.

Among the 58 individuals who donated blood at both time periods, the median concentrations of PFOS, PFOSAA, PFOA, PFHS, and M570 were statistically significantly higher in 1989 than in 1974 (Table 6). Among the 120 samples collected from different individuals in 1974 and 1989, PFOSAA, PFOA, and PFHS were significantly higher in 1989 than in 1974, adjusted for age and sex (Table 7). It was not possible to add smoking and education to the model because the iterations did not converge.

Because PFOSAA and M570 are fluorochemical residuals that may degrade to some unknown degree to PFOS, we examined the correlations between these compounds. In the median regression models for the 1989 data with PFOS as the dependent variable, the regression coefficient was 0.73 (*p* < 0.0001) for PFOSAA and 2.98 (*p* = 0.018) for M570. In contrast, no statistically significant associations were observed in 1974. We also examined the association between PFOS and PFOA because these two compounds were reported to be highly correlated in other studies (Olsen et al. 2003d, 2004a, 2004b). Spearman correlations between PFOS and PFOA were 0.53 (*p* < 0.0001) in 1974 and 0.60 (*p* < 0.0001) in 1989.

A food frequency questionnaire was completed by 51 of the 178 participants who provided blood samples in 1989. We evaluated these data to determine if there was an association between fast-food consumption and plasma PFOSAA concentrations because our hypothesis was that those who consumed more fast food may have been exposed to N-EtFOSE residuals that may be found in paper or package protectants applied to wrappings in take-out food operations. No statistically significant differences were observed for median plasma PFOSAA concentrations (metabolite of N-EtFOSE) for those who ate foods between once a week and once a day compared with less than once a week for hamburgers (2.8 vs. 4.1 ng/mL), fried chicken (3.4 vs. 5.7 ng/mL), pizza (3.4 vs. 3.5 ng/mL), and fried fish (3.6 vs. 3.6 ng/mL), respectively.

The results from statistical bootstrap analyses conducted to provide estimates of upper tolerance limits of the distributions are presented in Table 8. The upper tolerance limits represent the concentration of each fluorochemical below which the stated proportion of the population is expected to be found. Presented are the biased corrected estimates for the 90%, 95%, and 99% upper tolerance limits of the five fluorochemicals along with the upper limit (bound) from the 95% CI. For example, the bias-corrected estimate of the upper 95% tolerance limit for PFOS in 1974 was 73 ng/mL with an upper 95% confidence limit of 112 ng/mL.

**Figure 1:**
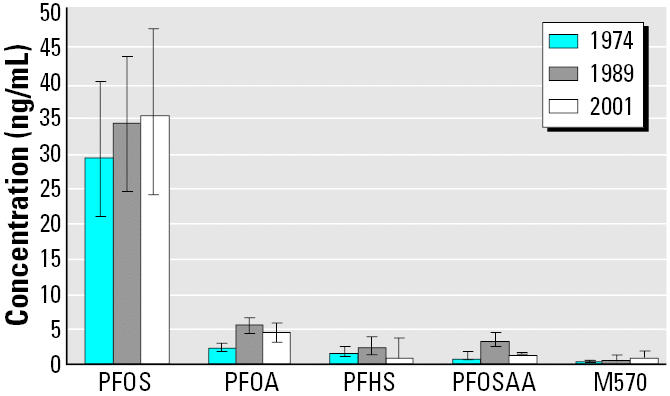
Median fluorochemical concentrations and IQRs for blood samples collected in Washington County, Maryland, from adults living in proximity in 1974 (*n* = 178 serum samples) and 1989 (*n* = 178 plasma samples) and in the county in 2001 (*n* = 108 serum samples; Olsen et al. 2003c).

Figure 1 shows the median and IQRs of the fluorochemical concentrations in the present study compared with the 2001 results reported for 108 Hagerstown area subjects (median age, 48 years; 55% male, 45% female) analyzed by Olsen et al. (2003c). Most samples were measured above the LLOQ in each year for PFOS and PFOA. GMs (and 95% CIs) for PFOS in 1974, 1989, and 2001 were 30.1 ng/mL (27.8–32.6), 33.3 ng/mL (31.1–35.6), and 35.3 ng/mL (31.8–39.1), respectively, with the 2001 GM statistically significantly different than the 1974 but not 1989 values. GMs (and 95% CIs) for PFOA in 1974, 1989, and 2001 were 2.1 ng/mL (1.9–2.2), 5.5 ng/mL (5.2–5.9), and 4.2 ng/mL (3.8–4.8), respectively, with each year being significantly different than the other years. It was not possible to adequately detect trends over this 27-year time period for PFHS, PFOSAA, and M570 because of the large number of samples in each year that were below the LLOQ and because, when compared with the present study, higher LLOQ values were used (< 2.1, < 2.8, and < 1.8 ng/mL, respectively) with the 2001 samples analyzed by Olsen et al. (2003c). Nevertheless, the median and IQRs appear not to have substantially increased (Figure 1).

## Discussion

Our data indicate that PFOS concentrations increased in this population between 1974 and 1989. Based on the 58 paired samples in the present study, the median PFOS concentration in the blood (serum or plasma) increased 25% from 1974 (median, 24.9 ng/mL) to 1989 (median, 33.2 ng/mL). For the nonpaired samples, a statistically nonsignificant 9% difference in median values was observed between 1974 (median, 32.8 ng/mL) and 1989 (median, 35.7 ng/mL). The change in the paired and nonpaired sample results are not directly comparable because the findings may be confounded by age. In 1974, age was lower for the paired samples compared with nonpaired samples (mean ± SD of 42.3 ± 12.3 vs. 47.8 ± 19.1, respectively). In 1989 age was significantly higher in the paired samples compared with the nonpaired samples (mean ± SD of 57.2 ± 12.6 vs. 48 ± 17.5, respectively). The distribution by sex was similar for the paired and nonpaired samples in each year. Because the paired sample group represents within-person change over time, unlike the nonpaired samples, and were chosen *a priori* to specifically examine this issue, we believe these data analyses support the inference of the occurrence of a change in PFOS concentrations in our study population between 1974 and 1989. This change in concentration may be due to increased exposure (consumer or environmental) and/or bioaccumulation. Production of POSF-derived products increased substantially (5-fold) between 1975 and 1989 (Santoro M, personal communication), and the number of applications for those products also increased, including the use in human food contact applications. PFOS concentrations were lower in the younger (< 40 years) age range in 1974 compared with the older age categories. This association was not observed in 1989. A speculative explanation could be that this younger age category in 1974 may have had less opportunity for potential exposure to some POSF-based product lines, environmental exposure, or even *in utero* exposure (Inoue et al. 2004) than the same age category 15 years later.

PFOS concentrations may not have increased since 1989 because the median values of the paired and nonpaired samples approximated the median value (36 ng/mL) of 108 samples of American Red Cross blood donors from the Hagerstown area that were collected in 2001 (Olsen et al. 2003c). These 108 Hagerstown area donors were comparable in age and sex to those individuals analyzed in 1974 and 1989. This median PFOS concentration among the Hagerstown donors was also similar to five other geographic locations measured by Olsen et al. (2003c). Statistical bootstrap estimates of the upper bound of the 95th percentile of PFOS in the 1974 and 1989 time periods do not indicate a shift in the upper tail of the distribution of serum PFOS concentrations when compared with the Hagerstown 2001 data. The upper bound estimate in the present study was 112 ng/mL in 1974 and 69 ng/mL in 1989, and was reported as 100 ng/mL based on 645 American Red Cross adult blood donors collected in 2001 by Olsen et al. (2003c). Although any explanation for the similar serum PFOS concentrations measured in 1989 and 2001 from residents living in proximity to Hagerstown would be speculative, one possibility could be the relatively consistent production of POSF that occurred between 1989 and 1999 (the year before 3M’s voluntary phase-out announcement) (Santoro M, personal communication). This might explain the relative steady-state serum concentrations in these populations that would have been obtained from various potential sources of exposure (consumer and environmental) during this time period. By comparison, total worldwide POSF production by 3M was reduced 20-fold in 2001 compared with 2000, the year of the company’s voluntary phase-out announcement.

Since the 1960s several studies have reported total organic fluorine concentrations in human blood from presumably non-occupationally exposed persons. Guy (1972), Singer and Ophaug (1979), and Taves et al. (1976) reported mean total organic fluorine concentrations of 30 (*n* = 65), 25 (*n* = 106), and 45 (*n* = 264) ppb, respectively. Using nuclear magnetic resonance spectroscopy, Taves (1968a, 1968b; Taves et al. 1976) determined organic fluorine was bound to albumin and tentatively identified a component of the organic fluorine as PFOA but also hypothesized that branching or the presence of a sulfonate was a possible interpretation of their findings. To compare measured PFOS concentrations in serum with total organic fluorine values, PFOS can be multiplied by the fraction of the molecular weight (0.65) contributed by the carbon-bound fluorine. If done for the median values reported in the present study (Tables 2–5), this yields 19 and 23 ppb organic fluorine for 1974 and 1989, respectively. This indicates that the 1974 serum PFOS results reported herein are consistent with the inference that PFOS was likely the predominant organofluorochemical in the serum at the time.

Whether measured in the paired or non-paired samples, there was a 4-fold increased concentration of PFOSAA in 1989 compared with 1974. This difference may be due to various factors, including as one hypothesis that the increase related to the commercialized use of the phosphate ester of N-EtFOSE–related products as protective coating on paper used for human food contact applications that began in 1974. However, we were unable to show an association between serum PFOSAA concentrations and frequency of fast-food consumption (where N-EtFOSE–related paper protectants might have been used) in 1989, albeit this was a small subset of study subjects. No increase in median serum PFOSAA concentrations was seen between the 1989 and 2001 data for this area (Figure 1).

M570 serum concentrations may have increased between 1974 and 1989 with 93% of the samples < LLOQ in 1974 compared with 61% < LLOQ in 1989. Median values remained the same between these 2 years. Most M570 samples measured in 2001 from the 108 Hagerstown blood donors were < LLOQ (measured at either 1.0 or 1.8 ng/mL depending upon the batch analysis) (Olsen et al. 2003c).

Because they are metabolized quickly and have relatively short serum elimination half-lives (Butenhoff J, personal communication), PFOSAA and M570 as serum concentrations should be considered markers for only recent nonoccupational exposure in the general population. This is unlike PFOS, PFOA, and PFHS, which have estimated serum elimination half-lives of several years (Burris et al. 2002). Because the commercialization of N-EtFOSE–related products for human food packaging applications did not commence until the mid-1970s, our data also suggest that human PFOS serum concentrations observed in 1974 were likely from either environmental origin and/or from N-MeFOSE–related products that were commercially introduced in the 1950s.

Our study data also indicate that serum PFOA may have also increased by a few nanograms per milliliter between 1974 and 1989 representing a 2-fold rise although the source was not determined. The subset analysis of the 58 paired samples supports this approximate 2-fold increase in serum PFOA concentrations since 1974. This trend may not have continued into the more recent time period because the median PFOA value was 4.5 ng/mL among the 108 Hagerstown, Maryland (Washington County), blood donors in 2001, similar to that measured in the present study in 1989.

We observed statistically significant correlations between PFOS and PFOA in both time periods. These were similar to the correlations (Pearson correlation coefficient ~ 0.7) reported in more recently collected blood samples from children, adults, and elderly in the United States (Olsen et al. 2003c, 2004a, 2004b). These associations between PFOS and PFOA are of interest because PFOA is not known to convert directly from PFOS (or vice versa). Whether these statistical associations are due to the presence of PFOA as a by-product in POSF-related production or to other nonrelated environmental exposures or consumer products (e.g., higher chain telomers) remains to be determined.

Several limitations could exist in this study. First, serum was the medium analyzed in 1974, versus plasma in 1989. The validated study of the analytical methods, however, provided comparable findings regardless of serum or plasma (Tandem Labs 1999, 2001a, 2001b). Conjugated forms of PFOSAA and M570, if they exist, were not accounted for in these analyses. Systematic biases were minimized between the 1974 and 1989 laboratory analyses because the samples were measured concurrently, although freezer storage time was different. Comparison of our 1974 and 1989 data with the American Red Cross adult blood donor data from Hagerstown, Maryland, collected in 2001 (Olsen et al. 2003c) allows for the potential for possible drift in laboratory results because of a different time of analysis, although the same analytical method and laboratory were used. Many samples were measured below the LLOQ of the assay for some of the analytes; thus, measures of central tendency for the population distributions were not exact. This was not the case, however, for PFOS for either time period or for PFOA, PFOSAA, and PFHS in 1989.

In conclusion, the present study was designed to assess the concentration trends of PFOS, PFOA, and five other fluorochemicals in blood samples from individuals in the general population who resided near Washington County, Maryland, between 1974 and 1989. Analysis of blood samples collected from the same 58 individuals indicated the median PFOS concentration increased 25% (~ 10 ng/mL). Among the same individuals, PFOSAA and PFOA increased a few nanograms per milliliter between 1974 and 1989, which resulted in a 2-fold increase in concentrations. A comparison with other regional data collected in 2001 did not suggest a continued increase in concentrations since 1989.

## Figures and Tables

**Table 1:** Demographic characteristics of study participants.

Characteristic	1974 (*n* = 178)	1989 (*n* = 178)	*p*-Value*^a^*
Age (years)			
Median (IQR)	47.5 (30–60)	52 (39–65)	0.007*^b^*
Sex [no. (%)]			
Male	87 (49)	86 (48)	
Female	91 (51)	92 (52)	0.92*^c^*
Education, years [no. (%)]			
< 12	61 (34)	38 (21)	
12	63 (35)	63 (35)	0.08
> 12	53 (30)	77 (43)	0.002
Missing data	1 (1)	0 (0)	—
Cigarette smoking [no. (%)]			
Never	85 (48)	90 (51)	
Former	51 (29)	52 (29)	0.88
Current	42 (24)	36 (20)	0.44

a Mixed logistic regression model that included paired and nonpaired samples.

b Age not significantly different when only nonpaired data analyzed.

c Logistic model included only nonpaired samples.

**Table 2:** Serum concentrations (ng/mL) of five fluorochemicals in 1974: summary data of 178 samples.

Analyte	LLOQ*^a^*	> LLOQ [*n* (%)]	Median (IQR)	GM (95% CI)
PFOS	3.9	178 (100)	29.5 (21.1–40.2)	30.1 (27.8–32.6)
PFOSAA	1.6	58 (33)	0.8 [LLOQ (1.6)–1.8]	1.2 (1.1–1.3)
M570	1.0	7 (4)	0.5 [< LLOQ (1.0)]*^b^*	0.5 (0.5–0.6)
PFOA	1.9	127 (71)	2.3 [< LLOQ (1.0)–3.0]	2.1 (1.9–2.2)
PFHS	1.4	112 (63)	1.6 [(< LLOQ (1.4–2.5)]	1.5 (1.3–1.6)

a Values < LLOQ were imputed as midpoint between zero and the LLOQ.

b All M570 values in the interquartile range were < LLOQ (1.0).

**Table 3:** Serum concentrations (ng/mL) of five fluorochemicals in 1974 by sex and age.

	By sex			By age			
Analyte	Male (*n* = 87) [median (IQR)]	Female (*n* = 91) [median (IQR)]	*p*-Value*^a^*	Age < 40 (*n* = 66) [median (IQR)]	Age 40–60 (*n* = 67) [median (IQR)]	Age > 60 (*n* = 45) [median (IQR)]	*p*-Value*^a^*
PFOS	32.8 (23.3–42.8)	25.3 (20.0–35.9)	0.01	24.8 (20.1–34.6)	31.7 (23.0–39.0)	35.3 (25.1–50.3)	0.02
PFOSAA	0.8 [< LLOQ (1.6)–1.9]	0.8 [< LLOQ (1.6)–1.8]	0.63	0.8 (0.8–2.0)	0.8 (0.8–1.7)	0.8 (0.8–1.6)	0.08
M570	0.5 [< LLOQ (1.0)]	0.5 [< LLOQ (1.0)]	0.75	0.5*^b^*	0.5*^b^*	0.5*^b^*	0.87
PFOA	2.4 (1.0–3.0)	2.3 (1.0–3.1)	0.53	2.2 (0.9–2.7)	2.4 (0.9–3.0)	2.4 (0.9–3.2)	0.39
PFHS	1.9 [< LLOQ (1.4)–3.1]	1.4 [< LLOQ (1.4)–2.2]	< 0.0001	0.7 [< LLOQ (1.4)–1.7]	1.9 (0.7–2.6)	2.2 (1.6–3.0)	< 0.0001

a Wilcoxon rank sum test.

b All M570 values in the IQR were < LLOQ (1.0).

**Table 4:** Plasma concentrations (ng/mL) of five fluorochemicals in 1989: summary data of 178 samples.

Analyte	LLOQ*^a^*	> LLOQ [*n* (%)]	Median (IQR)	GM (95% CI)
PFOS	3.9	178 (100)	34.7 (25.0–44.0)	33.3 (31.1–35.6)
PFOSAA	1.6	166 (93)	3.4 (2.5–4.7)	3.6 (3.2–4.0)
M570	1.0	68 (38)	0.5 [< LLOQ (1.0)–1.3]	0.8 (0.7–0.9)
PFOA	1.9	177 (99)	5.6 (4.4–6.7)	5.5 (5.2–6.9)
PFHS	1.4	145 (82)	2.4 (1.3–1.6)	2.5 (2.2–2.8)

a Values < LLOQ were imputed as midpoint between zero and the LLOQ.

**Table 5:** Plasma concentrations (ng/mL) of five fluorochemicals in 1989 by sex and age.

	By sex			By age			
Analyte	Male (*n* = 86) [median (IR)]	Female (*n* = 92) [median (IQR)]	*p*-Value*^a^*	Age < 40 (*n* = 45) [median (IQR)]	Age 40–60 (*n* = 67) [median (IQR)]	Age > 60 (*n* = 66) [median (IQR)]	*p*-Value*^a^*
PFOS	38.3 (31.7–50.0)	30.4 (21.3–41.0)	0.0003	33.3 (27.0–44.1)	33.6 (25.3–41.5)	35.1 (24.5–50.0)	0.99
PFOSAA	3.4 (2.6–4.6)	3.2 (2.5–4.9)	0.67	3.4 (2.6–5.2)	3.3 (2.5–4.7)	3.4 (2.3–4.6)	0.72
M570	0.5 [< LLOQ (1.0)–1.4]	0.5 [< LLOQ (1.0)–1.3]	0.21	0.5 [< LLOQ (1.0)–1.6]	0.5 [< LLOQ (1.0)–1.1]	0.5 [< LLOQ (1.0)–1.4]	0.23
PFOA	5.8 (4.8–6.8)	5.5 (3.6–6.7)	0.11	5.3 (3.6–6.3)	5.5 (4.4–6.8)	6.0 (4.8–7.2)	0.01
PFHS	2.8 (2.2–4.2)	2.1 [< LLOQ (1.4)–3.1]	0.003	1.8 [< LLOQ (1.4)–2.6]	2.5 (1.4–4.2)	3.0 (2.2–5.2)	0.0003

a Wilcoxon rank sum test.

**Table 6:** Changes in serum fluorochemical concentrations (ng/mL) from 1974 to 1989 in 58 paired samples.

Analyte	1974 median*^a^* (IQR)	1989 median*^a^* (IQR)	Percent difference*^b^*	*p*-Value*^c^*
PFOS	24.9 (20.5–34.4)	33.2 (24.8–41.1)	25	0.0003
PFOSAA	0.8 [< LLOQ (1.6)–2.0]	3.4 (2.5–5.0)	204	< 0.0001
M570	0.5 [< LLOQ (1.0)]	0.5 [< LLOQ (1.0)]	0	< 0.0001*^d^*
PFOA	2.3 (1.0–2.8)	5.6 (4.3–6.9)	162	< 0.0001
PFHS	1.5 [< LLOQ (1.4)–1.9]	2.1 (1.5–3.1)	57	< 0.0001

a Values < LLOQ were imputed as midpoint between zero and the LLOQ (LLOQ values are presented in Tables 2 and 4).

b Median percent difference for within-subject changes.

c *p*-Value from Wilcoxon signed-rank test for paired samples analysis.

d Although the median value for M570 is the same for both years, the *p*-value is based on 41% of paired samples increased to only 3% decrease (the rest remained the same).

**Table 7:** Changes in fluorochemical concentrations (ng/mL) from 1974 to 1989 in nonpaired samples adjusted for age and sex (*n* = 120 samples/year).

Analyte	1974 median*^a^* (IQR)	1989 median*^a^* (IR)	Percent difference*^b^*	*p*-Value*^c^*
PFOS	32.8 (21.9–45.0)	35.7 (25.3–45.8)	9	0.50
PFOSAA	0.8 [< LLOQ (1.6)–1.8]	3.4 (2.5–4.4)	324	< 0.0001
M570	0.5 [< LLOQ (1.0)]	0.5 [< LLOQ (1.0)–1.3]	0	1.0
PFOA	2.4 (1.0–3.0)	5.7 (4.4–6.7)	138	< 0.0001
PFHS	1.8 [< LLOQ (1.4)–2.6]	2.6 (1.8–4.2)	46	< 0.0001

a Values less than the LLOQ were imputed as midpoint between zero and the LLOQ (LLOQ values are presented in Tables 2 and 4).

b Percent median difference.

c *p*-Value from a median regression analysis adjusted for age and sex.

**Table 8:** Estimates (ng/mL) of upper tolerance limits and upper confidence limits for five serum fluorochemicals, 1974 and 1989.

		1974		1989	
Fluorochemical	Upper tolerance limit (%)	Estimate*^a^*	Upper limit of 95% CI	Estimate*^a^*	Upper limit of 95% CI
PFOS	90	57	68	59	62
	95	73	112	65	69
	99	140	178	82	123
PFOSAA	90	3	3	8	11
	95	4	5	14	29
	99	7	13	52	127
M570	90	LLOQ*^a^*	LLOQ*^a^*	2	3
	95	LLOQ*^a^*	LLOQ*^a^*	3	4
	99	3	5	5	6
PFOA	90	4	4	9	10
	95	5	6	11	13
	99	6	8	21	72
PFHS	90	4	4	8	10
	95	5	6	12	15
	99	7	11	33	65

a Estimated value at < LLOQ (1.0 ng/mL).
